# Evaluating a Change in Surgical Antibiotic Prophylaxis in Kidney Transplant Recipients

**DOI:** 10.7759/cureus.3433

**Published:** 2018-10-09

**Authors:** Katherine Bliven, Karisa Snow, Adrian Carlson, Sarah Yeager, Nicole Kenyon, Lonnie Smith, Crystal M Truax, Eryberto Martinez, Jeffrey Campsen

**Affiliations:** 1 Surgery, University of Utah, Salt Lake City, USA

**Keywords:** surgical wounds, human, kidney transplant, infection prophylaxis, antibiotic prophylaxis

## Abstract

The purpose of this study was to retrospectively evaluate if a change in practice from January 2013 to August 2015 affected the rate of surgical-site infections following kidney transplantation at the single academic medical center. More patients were found to have a surgical-site infection when surgical antibiotics were only given intra-operatively despite a lower incidence of risk factors identified in the literature when compared to the cohort who received antibiotics intra-op and post-op for 24 hours.

## Introduction

Kidney transplantation is a well-established and highly successful treatment for end-stage renal disease. In 2014, over 17,000 kidney transplants took place in the United States [1]. Despite its rate of success, multiple complications can arise from the transplant procedure. The rate of post-operative infections following a kidney transplant ranges from 1 to 56%, with one of the most common infections being surgical-site infections, which occur in up to 11% of patients [2]. Although surgical-site infections rarely lead to graft loss, they can lead to significant morbidity and prolonged hospitalization [3]. Multiple risk factors have been identified that increase the risk of acquiring a surgical site infection in the kidney transplant population including body mass index (BMI) greater than 27, immunosuppression regimen, older age, diabetes, cold ischemic time of more than 30 hours, duration of surgical procedure longer than 200 minutes and delayed graft function [3-6]. Diabetes, coronary artery disease and peripheral vascular disease are also literature reported risk factors for surgical-site infections in this population [3-6].

The risk of surgical-site infections related to immunosuppression may be affected by the intensity of immunosuppression, the receipt of these medications prior to transplant and certain agents pose a higher risk. The use of immunosuppressive medications prior to transplant is also a risk factor for surgical-site infections in kidney transplant recipients. Patients who receive more intense immunosuppressive regimens will likely be at a greater risk of developing a surgical-site infection. Mycophenolate mofetil (MMF) and mammalian target of rapamycin (mTOR) inhibitors are commonly used in maintenance therapy following a kidney transplant and are independent risk factors for surgical-site infections [2]. In addition, corticosteroids and mTOR inhibitors delay wound healing in multiple studies [6].

There currently is no consensus on the optimal perioperative prophylaxis regimen for renal transplant surgery [4, 7]. However, there is consensus that perioperative antibiotics are to be administered within 60 minutes of surgical incision and are not to last longer than 24 hours [2]. The American Society of Health-System Pharmacists (ASHP), the Infectious Diseases Society of America (IDSA), the Surgical Infection Society (SIS), and the Society for Healthcare Epidemiology of America (SHEA) jointly developed guidelines for antimicrobial prophylaxis in surgery. These joint guidelines have specific recommendations for kidney transplant surgery and state that cefazolin is the preferred antibiotic in this patient population (strength of evidence = A) [2]. The guidelines recommend that non-obese patients weighing less than 120 kg receive cefazolin 2 g IV within the one hour time frame and be re-dosed every four hours throughout surgery. The recommended dose of cefazolin is increased to 3 g IV in patients weighing greater than or equal to 120 kg. Clindamycin or vancomycin in combination with gentamicin, aztreonam, or a fluoroquinolone are alternatives for penicillin-allergic patients (strength of evidence = C) [2].

Surgical-site infections may lead to higher treatment cost, possible re-admissions, increased antibiotic use, possible surgical procedures, and increased risk of Clostridium difficile [4]. Less antibiotic exposure presents many advantages including reduced potential for resistant organisms, decreased health care costs and decreased potential for toxicity [4].

Prior to April 2014, the standard practice at our institution was to administer cefazolin 1 g IV intra-operatively followed by cefazolin 1 g IV every eight hours for 24 hours, post-operatively. In April 2014, the protocol changed to administer a single dose of cefazolin 1 g IV intra-operatively, and to no longer administer surgical prophylaxis post-operatively. If the surgical procedure lasts longer than four hours, both protocols required that the patient be re-dosed with the appropriate antibiotic(s) intra-operatively. Patients with a penicillin allergy were administered an alternative agent. The choice of alternative antibiotic in penicillin-allergic patients was not standardized at the institution and instead, antibiotic choice was at the discretion of the anesthesiologist or surgeon. Regardless of the antibiotic chosen, the duration of surgical antibiotic prophylaxis remained the same in both protocols.

The purpose of this study was to retrospectively evaluate if the change in practice affected the rate and classification of surgical-site infections following kidney transplantation at this single academic medical center.

## Materials and methods

The primary objective of this study was to determine if the change in surgical antibiotic prophylaxis in kidney transplant patients affected the rate and classification of surgical-site infections in this patient population, using the Centers for Disease Control (CDC) National Nosocomial Infection Surveillance System (NNIS) definition for surgical-site infections (Table 1) [8]. The secondary objectives of this study were to evaluate the effect of BMI and immunosuppression on the rate of surgical-site infections in kidney transplant recipients.

**Table 1:** CDC NNIS definition of a surgical-site infection. ^‡^ If the area around a stab wound becomes infected, it is not a surgical-site infection. It is considered a skin or soft tissue infection, depending on its depth. CDC: Centers for Disease Control; NNIS: National Nosocomial Infection Surveillance System.

Classification of surgical-site infections	Description	And requires one of the following
Superficial incisional surgical-site infection (SSI)	Infection occurs within 30 days after the operation AND Infection involves only skin or subcutaneous tissue of the incision Do not report the following conditions as surgical-site infection: Stitch abscess (minimal inflammation and discharge confined to the points of suture penetration) Incisional SSI that extends into the fascial and muscle layers (see deep incisional SSI)	Purulent drainage, with or without laboratory confirmation, from the superficial incision Purulent drainage, with or without laboratory confirmation, from the superficial incision Organisms isolated from an aseptically obtained culture of fluid or tissue from the superficial incision At least one of the following signs or symptoms of infection: Pain or tenderness, localized swelling, redness, or heat and superficial incision is deliberately opened by surgeon, unless incision is culture-negative Diagnosis of superficial incisional surgical-site infection by the surgeon or attending physician.
Deep incisional surgical-site infection	Infection occurs within 30 days after the operation AND Infection involves deep soft tissues (e.g., fascial and muscle layers) of the incision Notes: Report infection that involves both superficial and deep incision sites as deep incisional surgical-site infection Report an organ/space surgical-site infection that drains through the incision as a deep incisional surgical-site infection	Purulent drainage from the deep incision but not from the organ/space component of the surgical site A deep incision spontaneously dehisces or is deliberately opened by a surgeon when the patient has at least one of the following signs or symptoms: fever (>38°C), localized pain, or tenderness, unless site is culture-negative An abscess or other evidence of infection involving the deep incision is found on direct examination, during reoperation, or by histopathologic or radiologic examination Diagnosis of a deep incisional surgical-site infection by a surgeon or attending physician
Organ space surgical-site infection	Infection occurs within 30 days after the operation AND Infection involves any part of the anatomy (e.g., organs or spaces), other than the incision, which was opened or manipulated during an operation	Purulent drainage from a drain that is placed through a stab wound‡ into the organ/space Organisms isolated from an aseptically obtained culture of fluid or tissue in the organ/space An abscess or other evidence of infection involving the organ/space that is found on direct examination, during reoperation, or by histopathologic or radiologic examination Diagnosis of an organ/space surgical-site infection by a surgeon or attending physician

This study was a retrospective review of an observational cohort of patients who received a kidney transplant at a single academic medical center. A senior researcher reviewed all patients for surgical-site infections. Two independent researchers did all chart reviews manually. A third reviewer was available to clarify any questions or discrepancies found.

All recipients of a kidney transplant alone at the institution from January 1, 2013 to August 31, 2015 were eligible for inclusion. All recipients of any other organ at the same time as kidney transplant were excluded. Patients were divided into two groups: the Continuance group and the One-and-Done group. The Continuance group included 50 patients who received the historical surgical prophylaxis protocol of antibiotics given intra-operatively and continued for 24 hours post-operatively. The One-and-Done group included 50 patients who received one dose of antibiotics given intra-operatively only.

A power calculation indicated that over 400 patients were required in each group to detect a significant difference (assuming 11% incidence of surgical-site infection and a 5% decrease). It was not possible to achieve a study population of this size at the single academic medical center within a reasonable time frame, as this center averages approximately 80–100 kidney transplants annually. Rather, the investigators sought to look at trends in the data available within the institution.

## Results

The baseline characteristics of all patients included in this study are presented in Table 2. Most patients were male, the average age was 49 years (SD+18.8 years), most patients received tacrolimus, mycophenolate and prednisone for maintenance immunosuppression and there was a similar number of deceased-donor kidney transplants in each group. There was a higher use of antibody induction (Continuance, n = 32 vs One-and-Done, n = 24), incidence of delayed graft function (Continuance, n = 2 vs One-and-Done, n = 0), and more patients with a BMI greater than 27 (Continuance, n = 30 vs One-and-Done, n = 20) in the Continuance group. At the time of transplant, diabetes (Continuance, n = 16 vs One-and-Done, n = 11), coronary artery disease (Continuance, n = 9 vs One-and-Done, n = 6) and use of immunosuppression (Continuance, n = 10 vs One-and-Done, n = 5) were also more common in the Continuance group. Overall, there were more literature reported risk factors present in the Continuance group compared to One-and-Done group.

**Table 2:** Baseline characteristics of patients (n = 100). ESRD: End-stage renal disease; GN: Glomerulonephritis; BMI: Body mass index; CIT: Cold ischemic time.

	Continuance (n = 50)	One-and-Done (n = 50)
Age, average year (range year)	46.7 (24-73)	47.0 (22-73)
Male	58% (n = 29)	68% (n = 34)
Etiology of ESRD		
Alports	8% (n = 4)	2% (n = 1)
Chronic glomerulonephritis	24% (n = 12)	16% (n = 8)
Diabetes	22% (n = 11)	16% (n = 8)
Drug toxicity	10% (n = 5)	6% (n = 3)
Hypertension	16% (n = 8)	24% (n = 12)
Obstructive nephropathy	8% (n = 4)	6% (n = 3)
Other	12% (n = 6)	16% (n = 8)
Polycystic kidney disease	16% (n = 8)	10% (n = 5)
Retransplant	20% (n = 10)	14% (n = 7)
Reflux nephropathy	8% (n = 4)	12% (n = 6)
Renal dysplasia	-	4% (n = 2)
Unknown	-	4% (n = 2)
BMI > 27	60% (n = 30)	40% (n = 20)
Pre-transplant		
Diabetes	32% (n = 16)	22% (n = 11)
Coronary artery disease	18% (n = 9)	12% (n = 6)
Peripheral vascular disease	-	2% (n = 1)
Immunosuppression	20% (n = 10)	10% (n = 5)
Deceased donor transplant	60% (n = 30)	60% (n = 30)
Living donor transplant	40% (n = 40)	40% (n = 40)
Antibody induction		
Rabbit antithymocyte globulin	36% (n = 18)	24% (n = 12)
Alemtuzumab	28% (n = 9)	24% (n = 12)
None	36% (n = 18)	52% (n = 26)
CIT > 30 hours	-	-
Duration of operation > 200 minutes	4% (n = 2)	22% (n = 11)
Delayed graft function	4% (n = 2)	-

Within the first 30 days post-transplant, zero deep incisional and organ space surgical-site infections were found. Eight superficial incisional surgical-site infections were identified, two in the Continuance group and six in the One-and-Done group (p = 0.16, Fisher Exact Test). The overall infection rate in the Continuance group was 4% and 12% in the One-and-Done group. The risk factors present in patients with surgical-site infections are illustrated in Table 3.

**Table 3:** Risk factors present in patients with surgical-site infections. Y: Risk factor present; N: Risk factor not present; BMI: Body mass index; DM: Diabetes; CAD: Coronary artery disease; IS: Immunosuppression; ATG: Rabbit anti-thymocyte globulin; Vanco: Vancomycin; CFZ: Cefazolin; Alemtuz: Alemtuzumab; Clinda: Clindamycin; TX: Transplant.

Patient	Age	BMI > 27	Pre-TX DM	Pre-TX CAD	Pre-TX IS	Induction	Surgical prophylactic antibiotic
Continuance							
1	75	Y	Y	Y	N	ATG	Vanco 1 g
2	52	Y	Y	Y	N	ATG	CFZ 1 g
One-and-Done							
1	63	Y	Y	Y	N	None	CFZ 1 g
2	73	N	N	N	N	Alemtuz	CFZ 1 g
3	36	N	N	N	Y	ATG	Vanco 1 g
4	55	Y	N	N	N	None	Vanco 1 g
5	33	N	N	N	N	ATG	Clinda 600 mg
6	30	N	N	N	N	None	CFZ 1 g

When analyzing the cohort of patients with surgical-site infections further, four of the eight patients with surgical-site infections received surgical antibiotic(s) that were not part of the standard cefazolin regimens. The previously stated overall infection rate in Continuance group was 4% (2/50), and increased to 20% (1/5) if a non-cefazolin regimen was used. The overall surgical-site infection rate was 12% (6/50) in One-and-Done group, and increased to 30% (3/10) in patients who received a non-cefazolin regimen.

The rate of surgical-site infections was also increased among those patients who received cefazolin 1 g compared to the overall surgical-site infection rate, in both cohorts. No surgical-site infections occurred in patients who received 2 g or more of cefazolin intra-operatively (Continuance, n = 25 vs One-and-Done, n = 18) (Table 4).

**Table 4:** Analysis of infection rates in different antibiotic regimens. ^a ^Vancomycin 1 g used once in Continuance group and six times in One-and-Done group. ^b ^Single regimen used in 14/15 cases (cefoxitin 2 g + ertapenem 1 g used in one patient in One-and-Done group due to donor characteristics).

	Continuance	One-and-Done
Overall infection rate	4% (2/50)	12% (6/50)
Cefazolin 1 g intra-operatively	5% (1/20)	13.6% (3/22)
Cefazolin 2 g or more intra-operatively	0% (0/25)	0% (0/18)
Antibiotic other than cefazolin^a,b^	20% (1/5)	30% (3/10)

The choice of antibiotic prophylaxis among patients with surgical-site infections is presented in Table 3. This antibiotic choice was determined by the individual anesthesiologist or surgeon, and many patients who did not receive cefazolin, largely due to allergies, received a single dose of vancomycin 1 g and not dual therapy as recommended by the clinical practice guidelines for surgical prophylaxis.

Fifty percent of patients (n = 4) with a surgical-site infection had a BMI greater than 27. The median BMI among patients without a surgical-site infection was 27.1 compared to 26.7 in those with a surgical-site infection.

The differences among the use of immunosuppression prior to transplant and immunosuppressive regimen used during transplant for the Continuance group and One-and-Done are available in Table 2. Over 60% of the patients with a surgical-site infection received antibody induction and 13% were on immunosuppression prior to transplant. Eleven percent of patients in the Continuance group and 17% in the One-and-Done group received rabbit anti-thymocyte globulin for induction immunosuppression and developed a surgical-site infection. None of the patients who received alemtuzumab in the Continuance group versus 8.3% in the One-and-Done group developed a surgical-site infection. Maintenance immunosuppression with tacrolimus, mycophenolate and prednisone was similar between the two groups.

## Discussion

We predicted that the Continuance group would have a higher incidence of surgical-site infections because more literature-reported risk factors were present at baseline. However, this is not what was observed in this small cohort. More patients were found to have a surgical-site infection when surgical antibiotics were only given intra-operatively (One-and-Done group), despite a lower incidence of risk factors identified in the literature when compared to the cohort who received antibiotics intra-op and post-op for 24 hours (Continuance group). It is possible that the patients included in this small cohort have additional risk factors that have not been reported in the literature.

Additionally, there was an increased incidence of surgical-site infections when a non-cefazolin based regimen was used. The majority of the non-cefazolin regimens used a single antibiotic, such as vancomycin 1 g or clindamycin 600–900 mg. This suggests that the use of a single non-penicillin regimen is insufficient to prevent surgical-site infections in this patient population and dual therapy should be used, as recommended by the guidelines (Table 5). All the results support the recommendations outlined in the clinical practice guidelines for antimicrobial prophylaxis in surgery.

**Table 5:** Updated surgical antibiotic prophylaxis for kidney transplant recipients at single center.

	Preferred antibiotic	True penicillin-allergy
<60 kg	Cefazolin 1.5 g IV	Clindamycin 900 mg IV + levofloxacin 500 mg IV
60–120 kg	Cefazolin 2 g IV	Clindamycin 900 mg IV + levofloxacin 500 mg IV
>120 kg	Cefazolin 3 g IV	Clindamycin 900 mg IV + levofloxacin 500 mg IV

The incidence of surgical-site infections increased when cefazolin 1 g was used in either group. However, when cefazolin 2 g or more was used, as recommended by the guidelines, no surgical-site infections occurred. This suggests that the increased incidence of surgical-site infections was more likely due to the cefazolin dose rather than the duration.

When evaluating the impact of BMI on surgical-site infections, we expected that patients with a higher BMI would acquire more surgical-site infections, because a BMI greater than 27 is a literature-reported risk factor. In our study, the median BMI for patients with surgical-site infections (26.7) was below the median BMI for patients without surgical-site infections (27.1). It is possible that this reflected the small sample size of our study. However, 50% of patients with a surgical-site infection had a BMI greater than 27. BMI greater than 27 has been validated as an independent risk factor for surgical-site infections [5]. Lynch et al. and Pham et al. demonstrated that overweight and obese patients are at higher risk for acquiring a surgical-site infection [6, 7]. Lynch et al. included 869 patients (351 with BMI > 30). Most surgical-site infections were superficial and significantly more surgical-site infections occurred in the obese group (non-obese, 10.5% vs obese 23.7%; p < 0.001) [7].

Patients with more intense immunosuppression and immunosuppression prior to transplant, were expected to acquire more surgical-site infections. Nearly 63% of the eight patients with surgical-site infections did receive antibody induction (Table 3). The maintenance regimens utilized were similar in both groups (i.e., tacrolimus, mycophenolate) and steroid continuance occurred in 100% of the patients in the Continuance group compared to 98% in the One-and-Done group. In contrast, more patients in the Continuance group were on immunosuppressive medications prior to transplant compared to the One-and-Done group (20% in the Continuance group versus 10% in the One-and-Done group). The skewed patient characteristics in this study are not enough to suggest that pre-transplant immunosuppression is not a risk factor for surgical-site infections. Our study does suggest that an inadequate dose of prophylactic antibiotics can put patients at an increased risk for such infections.

There are notable limitations of this study to address. First, this study was not powered to detect a statistically significant difference for surgical-site infection rates, because over 800 patients would need to be included, based on 11% surgical-site infection rate. The volume of kidney transplants at this single academic medical center could not support a population of this size in the given time frame. In addition, there may have been differences in the baseline characteristics of the two groups, as the two groups because they were not matched. Although this is a limitation, the result of fewer surgical-site infections found in the group with more risk factors suggests the unmatched baseline characteristics did not sway the results. The results presented may have limited external validity because this was a small cohort at an individual institution.

Strengths of this study include the patient sample – it was a true representation of the population at hand. Therefore, the results were truly applicable to the sole institution and exhibited strong internal validity. Another strength was the method of chart review; the use of two consistent reviewers with one referee minimized bias.

## Conclusions

The results from this small retrospective review demonstrate that cefazolin 1 g, administered intra-operatively only, is insufficient to prevent surgical-site infections in kidney transplant recipients. It also suggests that a single agent, such as clindamycin or vancomycin 1 g alone, does not protect kidney transplant recipients from surgical-site infections. Therefore, we implemented a change in our protocol in accordance with the clinical practice guidelines for antimicrobial prophylaxis in surgery. Cefazolin 2 g (3 g in patients greater than 120 kg) is now the preferred regimen at out institution. Clindamycin in combination with levofloxacin is the preferred alternative regimen for use in penicillin-allergic patients. Less antibiotic exposure is advantageous because it can reduce the potential for resistant organisms, decrease health care costs and lessen the potential for toxicity. It also demonstrates the applicability of guideline-supported change in transplant, rather than a continuation of what has been perceived to work in the past. After looking at our data, we have the opportunity to standardize the dose to minimize surgical-site infections in the future.
